# Genome Sequence of *Oxalobacter formigenes* Strain HC-1

**DOI:** 10.1128/genomeA.00533-17

**Published:** 2017-07-06

**Authors:** Marguerite Hatch, Milton J. Allison, Fahong Yu, William Farmerie

**Affiliations:** a Department of Pathology, Immunology and Laboratory Medicine, University of Florida, College of Medicine, Gainesville, Florida, USA; b Iowa State University, Ames, Iowa, USA; c Interdisciplinary Center for Biotechnology Research, University of Florida, College of Medicine, Gainesville, Florida, USA

## Abstract

The lack of *Oxalobacter formigenes* colonization of the human gut has been correlated with the formation of calcium oxalate kidney stones and also with the number of recurrent kidney stone episodes. Here, we present the genome sequence of HC-1, a human strain isolated from an individual residing in Iowa, USA.

## GENOME ANNOUNCEMENT

An anaerobe, with a substrate-specificity for oxalate, was isolated from human and other animal feces and a new genus and species *Oxalobacter formigenes*, was established (1). Individuals forming oxalate kidney stones who are *Oxalobacter*-negative have significantly higher urinary oxalate and stone episodes correlate with the lack of *Oxalobacter* (2). Colonization of a mouse model of the genetic disease primary hyperoxaluria, type 1 with *Oxalobacter* resulted in a normalization of both hyperoxaluria and hyperoxalemia exhibited in noncolonized counterparts (3, 4). Since a human strain, HC-1, was tested in some small human clinical trials (5–7), the present study was undertaken to determine the complete genome sequence of the HC-1 strain which was archived in the Hatch laboratory, notated as HC-1^MH^, since 2011.

A genomic DNA library was prepared following the protocol specified by Pacific Biosciences (Menlo Park, CA). Briefly, genomic DNA was sheared to an average fragment length of 20 kb, using the SAGE ELF (Sage Science, Beverly, MA), end-repaired, and single-molecule real-time (SMRT) bell oligonucleotide adaptors blunt-end ligated to construct a DNA fragment library for sequencing on the Pacific Biosciences RSII platform. A single SMRT cell produced a total of 1.58 Gb in 93,480 polymerase reads having an *N*_50_ of 19.7 kb and a subread *N*_50_ is 9.9 kb. The HC-1 genome was assembled using HGAP version 3 (8), and annotated using RAST (http://rast.nmpdr.org) (9–11).

The complete HC-1^MH^ genome contains a single contig of 2,468,871 bp and has an average G+C content of 49.6%. A total of 2,599 genes were annotated by RAST, including 47 tRNAs, 7 ribosomal RNAs, and 2,545 predicted coding sequences (CDSs). RAST annotation assigns 1,062 (42%) of the 2,545 HC-1 CDSs as members of 336 categorized subsystems. Subsystems are defined as a set of functional roles implementing specific biological process or structure (12). In general, subsystems may be considered biological pathways. The most abundant subsystem classifications include 203 genes involved in protein metabolism; 169 involved in metabolism of cofactors, vitamins, prosthetic groups, and pigments; 205 in amino acid and derivative metabolism; and 108 in carbohydrate metabolism. A total of 1,483 CDSs (58%) are not assigned to specific subsystems.

The annotated HC-1^MH^ genome was compared to *O. formigenes* CC13 (NCBI accession no. NZ_ACDQ00000000) and *O. formigenes* HOxBLS (accession no. NZ_ACDP00000000). At the protein level, 2,473 of 2,545 (97%) HC-1 CDSs have greater than 99% identity with CDSs identified in CC13. The genome of HC-1^MH^ contains 54 CDSs not present in CC13, the majority of which (42 CDSs) are identified as hypothetical proteins. The remaining 12 CDSs identified in HC-1^MH^ but absent from CC13 largely represent phage-associated proteins, primarily clustered in a ~35 kb region of the HC-1^MH^ genome. Only 260 (10%) HC-1^MH^ CDSs share greater than 90% amino acid identity with HOxBLS CDSs. Compared to HOxBLS, 713 CDSs appear exclusively in the HC-1^MH^ genome, of which 533 are annotated as hypothetical proteins and the remaining 180 CDS annotations include proteins characterized as ABC and other transporters, bacteriophage-related proteins, transcriptional regulators, large subunit ribosomal proteins, and a small cluster of clustered regularly interspaced short palindromic repeat (CRISPR)-associated proteins.

### Accession number(s).

This genome sequencing project was deposited in GenBank under accession no. CP018787. The version described is the first version.
